# Chemical Composition and Attractant Activity of Volatiles from *Rhus potaninii* to The Spring Aphid *Kaburagia rhusicola*

**DOI:** 10.3390/molecules25153412

**Published:** 2020-07-28

**Authors:** Xiang Zhu, Li Li, Tom Hsiang, Yuping Zha, Zhixiong Zhou, Ran Chen, Xian Wang, Qinglai Wu, Junkai Li

**Affiliations:** 1 Engineering Research Center of Ecology and Agricultural Use of Wetland, Ministry of Education, College of Agriculture, Yangtze University, Jingmi Road 88, Jingzhou 434025, China; cjdxnxyzx@sina.com (X.Z.); lilyfight109@163.com (L.L.); zhouzhixiong0227@163.com (Z.Z.); changdachenran@163.com (R.C.); hanks_wong@163.com (X.W.); wql106@163.com (Q.W.); 2 Institute of Pesticides, Yangtze University, Jingmi Road 88, Jingzhou 434025, China; 3 Hubei Engineering Technology Center for Pest Forewarning and Management, College of Agriculture, Yangtze University, Jingmi Road 88, Jingzhou 434025, China; 4 School of Environmental Sciences, University of Guelph, Guelph, ON, Canada N1G 2W1; thsiang@uoguelph.ca; 5 Institute of Forest Protection, Hubei Academy of Forestry, Wuhan 430075, Hubei, China; zhayuping@163.com; 6 Institute of Entomology, College of Agriculture, Yangtze University Jingmi Road 88, Jingzhou 434025, China

**Keywords:** *Kaburagia rhusicola* Takagi, *Rhus potaninii* Maxim, chemical composition, attractant activity

## Abstract

*Rhus potaninii* Maxim, a type of sumac, is an economically important tree widely cultivated in mountainous areas of western and central China. A gall, called the bellied gallnut, induced by the aphid, *Kaburagia rhusicola* Takagi, is important in the food, medical, and chemical industries in China. Volatiles from *R. potaninii* were found to attract *K. rhusicola*, but little is known about them. The chemical composition of these volatiles was investigated using GC–MS analysis and Y-tube olfactometer methods. Twenty-five compounds accounting for 55.3% of the volatiles were identified, with the highest proportion of 1-(4-ethylphenyl)ethanone (11.8%), followed by 1-(4-hydroxy-3-methylphenyl)ethanone (11.2%) and *p*-cymen-7-ol (7.1%). These findings provide a theoretical basis for the preparation of attractants and could eventually lead to increased bellied gallnut yield.

## 1. Introduction

The bellied gallnut is an insect gall induced by *Kaburagia rhusicola* Takagi (Hemiptera: Aphidoidea: Pemphigidae: Eriosomatinae) parasitic on the leaves of *Rhus potaninii* Maxim (Anacardiaceae), commonly known as gallnut trees [1,2]. As a characteristic product of Chinese traditional forestry, bellied gallnut has the effect of heat-clearing, detoxification, blood-cooling, and hemostasis, and is widely used in traditional folk medicine, with efficacious therapeutic effects and few known side effects [3]. In addition, the tannic acid, gallic acid, and pyrogallic acid produced from the gallnut as a raw material are widely used in foods [4], medicines [3], dyes [5,6], industry (metallurgy, electronics industry, chemical), and other fields [7,8,9]. Due to its large size and high tannin content in biological resources, bellied gallnut has attracted much attention among various Chinese gallnuts [10]. With the growing demand for bellied gallnut in China and abroad, there has been an increasing research focus on it.

These studies have mainly focused on the cultivation of *R. potaninii* [11], the biology and ecology of gall aphids [12], the extraction and analysis of chemical components of bellied gallnut, and its clinical application and pharmacology [13,14,15]. The lifecycle of the galling aphid, *K. rhusicola* has recently been reviewed [16]. This insect is a typical transhominal parasitic insect, and in the early spring, aphids will migrate to their summer host, *R. potaninii*, for sexual reproduction and will induce the production of the gallnut. Subsequently, autumn migrant aphids will emerge from the bellied gallnut and fly to the winter host *Eurohypnum leptothollum*, thus completing the lifecycle. As a result, the best harvest time of gallnuts is in late summer and early autumn, when the gall is fully grown but the aphids inside have not penetrated the gall wall. Freshly picked gallnuts should be blanched in boiling water in time, and when the surface turns from yellowish brown to gray, immediately removed from the water and dried in the sun or low fire. Studies have shown that the volatile substances of the spring host plant play a pivotal role in the long-distance location of insects, especially in the host transfer period [17,18]. *K. rhusicola* has strong host plant specificity [16], which suggests that *R. potaninii* may release particular volatiles that attract *K. rhusicola*.

In this study, volatiles from host plants of *R. potaninii* were prepared by headspace collection, and after characterization, the attractant activity of these volatiles to *K. rhusicola* in Y-tube olfactometers were evaluated. The goal of this study was to increase our understanding of the chemical composition of *R. potaninii* volatiles that attract *K. rhusicola*, and to provide a basis for the preparation of corresponding galling aphid attractants with the eventual goal of increasing bellied gallnut production and yield.

## 2. Materials and Methods

### 2.1. Plants and Insects

A summer host plant, *R. potaninii* is a thermophilic and drought-tolerant tree species and is favored by loam and sandy loam below 1,000 m for cultivation. *R. potaninii* trees were cultivated in a nursery in Desheng, Hubei Province (32°27′ N, 109°51′ E; 580 m above sea level) from cuttings of three-year-old trees. The plants were authenticated by Dr Jingyuan Chen, a senior research fellow at Forest Protection Institute, Hubei Academy of Forestry, Wuhan, Hubei Province, China. The spring migrant galling aphids, *K. rhusicola* specimens were collected from the winter host *E. leptothollum* around the *R. potaninii* trees from Desheng, Hubei Province, China. The collected galling aphids were then carefully transferred and stored in glass culture dishes (150 mm diam., 25 mm high) filled with leaves of the winter host for later use.

### 2.2. Chemicals and Instruments

Solvents of analytical grade were purchased from commercial suppliers (Tianjin Kemiou Chemical Reagent Co., Ltd., Tianjin, China). The volatiles from the branches of *R. potaninii* were collected by QC-1S atmospheric sampler (Beijing Municipal Institute of Labour Protection, Beijing, China), and the gas flow rate was monitored by a glass type rotameter (Yuyao Yinhuan Flow meter Co., Ltd., Yuyao, China). The collected volatiles were analyzed with an Agilent 7890A-5975C Gas Chromatography-Mass Spectrometer (GC-MS) (Agilent Technologies, Inc. Santa Clara, CA, USA) with a DB-WAX column (30 m × 250 µm × 0.5 µm film thickness, Agilent Technologies; Agilent Technologies, Inc. Santa Clara, CA, USA).

### 2.3. Collection of Volatiles

The method of volatile collection from *R. potaninii* was similar to that reported in the literature [19,20]. In late February, during the peak period when the *K. rhusicola* was migrating from the winter host plant *E. leptothollum* to their summer host plant *R. potaninii*, 0.5 kg of branches of similar size with no visible insects nor disease damage were collected from the host plant *R. potaninii*, and then enclosed full in a 0.5 × 0.6 m plastic bag (Reynolds Oven bag, Lake Forest, IL, USA). An adsorbent trap consisting of a borosilicate glass tube (20 mm diameter, 300 mm length) containing 200 mg Porapak Q adsorbent (100–120 mesh, ANPEL Laboratory technologies Inc., Shanghai, China) was connected to one side of the bag, and an activated charcoal filter was on the opposite side of the bag. The filtered air was pumped into the bag, and then the air with plant volatiles was drawn through the adsorbent trap by a membrane pump at a rate of 500 mL/min for 6 h. After collection, the Porapak Q adsorbent was immediately washed with 2 mL HPLC-grade dichloromethane, and then the eluate was transferred to a 2 mL sample bottle and stored at −20 °C until analyses.

### 2.4. Attractant Activity

**Figure 1:**
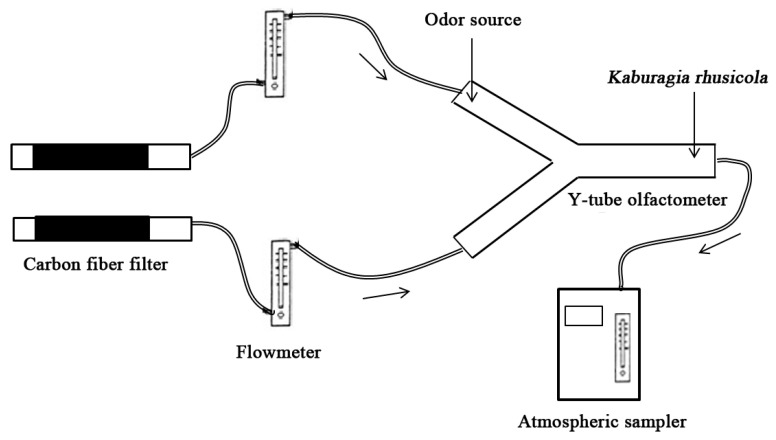
Device of Y-shape olfactometer test.

The attractiveness to *K. rhusicola* of volatiles from *R. potaninii* was measured in a Y-olfactometer. The collected volatile samples were used as odor sources, and the experimental device was set up as shown in Figure 1. The Y-tube olfactometer was made of a colorless and transparent glass tube, consisting of a base tube (70 mm i.d., 50 mm length) with two arms (40 mm i.d., 180 mm length) at 75°. The two arms of the “Y” tube were connected with a carbon fiber filter through soft PVC plastic tubing to purify the air stream. The PVC plastic tubes and the Y-olfactometer were connected by a perforated glass stopper, and a flowmeter was connected in the middle to measure the air flow rate. The atmospheric sampler was connected to the base tube of the Y-tube olfactometer through soft PVC plastic tubing to provide a continuous air stream. During measurement, volatile samples (10 µL) were dripped onto filter paper with a pipette and placed in the air source position of one arm of the Y-tube, and the solvent (dichloromethane) samples (10 µL) without volatiles were placed on the other arm as a control in the same manner. Subsequently, 50 galling aphids previously collected were quickly placed on the base of the Y-tube, and then air was pumped at a flow rate of 200 mL/min while each set was observed for 15 min. Aphids that stayed at the base tube of the Y-tube olfactometer were considered unresponsive, and aphids that entered the arm of the Y-tube were considered selective. The experiment was repeated three times. After each test, the sample and control were exchanged at the position of the arms, and the olfactory apparatus was cleaned with 95% ethanol. All statistical analyses were performed using IBM SPSS Statistical Version 18 (SPSS Inc., Chicago, IL, USA).

### 2.5. Chemical Composition Identification

The analyses of collected volatiles from *R. potaninii* were performed using an Agilent 7890A-5975C Gas Chromatography-Mass Spectrometer (GC-MS) equipped with a DB-WAX column with splitless injection. The analytical conditions of the GC-MS were as follows: the initial oven temperature was maintained at 40 °C for 1 min, increased by 5 °C/min to 170 °C held for 2 min, raised at a rate of 8 °C/min up to 230 °C, and then held at this temperature for 5 min. The inlet temperature was set to 230 °C, and the transfer line was at 250 °C. Injections (5 μL) were made using an auto-sampler in splitless injection mode, under electron impact (EI, 70 eV) and within the scan range of 30–300 amu. Helium gas was the carrier at a flow rate of 1 mL/min. Volatiles were identified using a GC-MSD chemical workstation data-processing system (Agilent) by comparing their mass spectra with data from the equipment database (NIST 05) and authenticated standards.

## 3. Results and Discussion

### 3.1. Attractant Activity

To evaluate the attractant activity to the spring migrant aphid *K. rhusicola* of volatiles released by the plant *R. potaninii*, the behavioral responses of *K. rhusicola* to volatiles were investigated with a glass Y-tube olfactometer. The results showed that volatiles of *R. potaninii* attracted significantly higher numbers of *K. rhusicola* than control receiving solvent treatment. The results of the preliminary behavioral responses are shown in Table 1. Compared with the 14.0 ± 1.2% selection rate of treatment of solvent control, the selection rate of *K. rhusicola* to volatiles was as high as 69.3 ± 2.4%, showing an obvious attraction effect, from which it could be concluded that volatiles play an important role in the process of *K. rhusicola* finding and locating the *R. potaninii*. However, it is not clear which specific chemicals and their proportions play a role in this process.

### 3.2. Characterization of Volatiles

The chemical composition of the volatiles from *R. potaninii* collected from the headspace is shown in Table 2. The peak area normalization method was used to process the ChemStation database to obtain the relative percentage content of each chemical component. GC–MS analysis of the volatiles revealed the presence of 25 compounds, representing 55.3% of the total relative content of the volatiles. It can be seen that the constituents of volatile compounds were aromatic hydrocarbon, alkenes, alkanes, aldehydes, ketones, and alcohols, and that the most abundant components were 1-(4-ethylphenyl)ethanone (11.8%), 1-(4-hydroxy-3-methylphenyl)ethanone (11.2%), *p*-cymen-7-ol (7.1%), 2-ethylhexan-1-ol (3.35%), and 3-ethylbenzaldehyde (3.32%). However, the amounts of the remaining 20 identified compounds were between 0.29% to 2.53%. Volatiles from plants have a number of alkene, aldehyde, and ketone components in common, but the relative amounts may differ [21]. The diversity and proportion of volatile components in different plants are important factors for the selection and location of insect feeding [22].

There is currently extensive research on the relationship between aphids and volatile components of host plants. 1-(4-ethylphenyl)ethanone was identified in the volatiles of four different host plants of aphids [23]. Visser and Fu-shun reported that the volatile compounds from the host plants of grain aphid and rose-grain aphid contain a trace amount of benzaldehyde with attractive activity [24], and similar combinations of ethylbenzene, tetradecane, and naphthalene were found in a soybean aphid-induced plant volatile of soybean [25]. Studies have shown that general plant odors can cause aphid electroantennogram (EAG) responses, while enols and enals can cause a greater response [26]. In this study, using Y-shape olfactometer and GC-MS methods, the attractant activity of volatiles to galling aphids was determined, and more than 20 volatile compounds that may play an important role in attracting *K. rhusicola* to *R. potaninii* were initially identified. The localization, feeding, and other behavioral responses of insects depend critically on their sophisticated and sensitive chemoreception system to recognize and distinguish a variety of semiochemical signals from the environment [27]. Odorant binding proteins (OBPs) of different insect species commonly need to combine with unique combinations of chemicals to produce corresponding behavioral responses in the process of olfactory sensing [28]. Thus, the components and proportion of the volatile compounds playing a decisive role during aphid migration to host trees need further investigation.

## 4. Conclusions

In conclusion, we identified 25 compounds, accounting for almost 55.3% of the total volatile content collected from *R. potaninii*. The volatiles released by *R. potaninii* exhibited an obvious attraction effect on the spring migrant aphid, *K. rhusicola.* Further studies can be carried out to determine which components and proportions of the volatile compounds play decisive roles attracting spring migrant aphids to host trees. These findings provide us with a firmer basis for the preparation of corresponding galling aphid attractants, with an eventual goal of increasing bellied gallnut yield.

## Figures and Tables

**Table 1:** Behavioral responses of *Kaburagia rhusicola* to *R. potaninii* volatiles or the control solvent dichloromethane in three experiments with 50 insects in each experiment after 15 min of exposure.

Treatment	Set 1	Set 2	Set 3	Selection Rate (%)
**Volatiles**	37	34	33	69.3 ± 2.4 a ^a^
**Solvent**	6	8	7	14.0 ± 1.2 b

^a^ Letters following the mean percentage (±SE) denote significant differences (one-way ANOVA, and *t*-test for two samples with *p* < 0.05), based on three replicates per experimental set.

**Table 2:** Volatile components of the branches of *R. potaninii* tree.

No.	Retention Time (min)	Compound	Mol. Weight	Molecular Formula	Class of Compound	Relative Content (%)
1	9.088	Ethylbenzene	106	C_8_H_10_	Arene	1.75 ± 0.28 ^a^
2	9.292	*p*-Xylene	106	C_8_H_10_	Arene	0.84 ± 0.10
3	12.915	Styrene	104	C_8_H_8_	Alkene	1.26 ± 0.18
4	13.883	Octanal	128	C_8_H_16_O	Aldehyde	2.02 ± 0.12
5	16.798	Tetradecane	198	C_14_H_30_	Alkane	0.26 ± 0.05
6	16.910	Nonanal	142	C_9_H_18_O	Aldehyde	1.14 ± 0.12
7	18.169	1-Ethyl-3-vinylbenzene	132	C_10_H_12_	Alkene	0.55 ± 0.10
8	18.461	1-Ethyl-4-vinylbenzene	132	C_10_H_12_	Alkene	0.99 ± 0.16
9	19.546	Pentadecane	212	C_15_H_32_	Alkane	0.66 ± 0.15
10	19.627	2-Ethylhexan-1-ol	130	C_8_H_18_O	Alcohol	3.35 ± 0.19
11	20.526	Benzaldehyde	106	C_7_H_6_O	Aldehyde	0.42 ± 0.03
12	20.663	1,2,3,4-Tetrahydronaphthalene	132	C_10_H_12_	Arene	0.35 ± 0.15
13	21.798	Longifolene	204	C_15_H_24_	Tricyclic sesquiterpenes	2.53 ± 0.17
14	23.845	Acetophenone	120	C_8_H_8_O	Ketone	0.50 ± 0.04
15	25.265	3-Ethylbenzaldehyde	134	C_9_H_10_O	Aldehyde	3.32 ± 0.28
16	26.003	Benzaldehyde, 4-ethyl-	134	C_9_H_10_O	aldehyde	2.07 ± 0.26
17	26.010	Isophthalaldehyde	134	C_8_H_6_O_2_	Aldehyde	1.32 ± 0.27
18	26.078	Naphthalene	128	C_10_H_8_	Arene	0.38 ± 0.04
19	28.181	1-(4-Ethylphenyl)ethanone	148	C_10_H_12_O	Ketone	11.84 ± 1.79
20	28.937	4-Vinylbenzoic acid	148	C_9_H_8_O_2_	Acid	0.50 ± 0.11
21	29.167	5-Allyl-1,3-benzodioxole	162	C_10_H_10_O_2_	Alkene	0.34 ± 0.05
22	31.319	*p*-Cymen-7-ol	150	C_10_H_14_O	Aromatic alcohol	7.08 ± 1.23
23	31.698	1-(4-hydroxy-3-methylphenyl)ethanone	150	C_9_H_10_O_2_	Ketone	11.20 ± 1.39
24	36.040	2,3-dimethylphenol	122	C_8_H_10_O	Phenol	0.29 ± 0.08
25	38.757	1,1′-(1,4-phenylene)diethanone	162	C_10_H_10_O_2_	Ketone	0.40 ± 0.06
		Total				55.33 ± 4.64

^a^ The volatile sample collected had three replicates (Mean ± SD).
